# Genetic Polymorphisms as Predictors of Survival in Breast Cancer: Future Lessons in Historical Data

**DOI:** 10.7759/cureus.21410

**Published:** 2022-01-19

**Authors:** Maria Leitão, Sarah Lopes, Deolinda Pereira, Rui Medeiros, Claudia Vieira

**Affiliations:** 1 Medical Oncology, Portuguese Institute of Oncology, Porto, PRT; 2 Molecular Oncology and Viral Pathology Group, Molecular Oncology and Viral Pathology Group, Instituto Português de Oncologia (IPO) Porto Research Center (CI-IPOP) Portuguese Oncology Institute of Porto (IPO Porto), Porto, PRT; 3 Medical Oncology, Abel Salazar Institute for the Biomedical Sciences (ICBAS), Porto, PRT; 4 Molecular Oncology, Molecular Oncology and Viral Pathology Group, Instituto Português de Oncologia (IPO) Porto Research Center (CI-IPOP) Portuguese Oncology Institute of Porto (IPO Porto), Porto, PRT; 5 Molecular Oncology, Abel Salazar Institute for the Biomedical Sciences (ICBAS), Porto, PRT; 6 Faculdade de Medicina da Universidade do Porto (FMUP), Faculty of Medicine University of Porto, Porto, PRT; 7 Centro de Estudos em Biomedicina (CEBIMED), Faculty of Health Sciences Fernando Pessoa University, Porto, PRT; 8 Research Department, Portuguese League Against Cancer—Regional Nucleus of the North (Liga Portuguesa Contra o Cancro—Núcleo Regional do Norte), Porto, PRT; 9 Molecular Oncology and Viral Pathology Group, Instituto Português de Oncologia (IPO) Porto Research Center (CI-IPOP) Portuguese Oncology Institute of Porto (IPO Porto), Porto, PRT

**Keywords:** genetic polymorphism, oprm1 genotype polymorphism, comt genotype polymorphism, breast cancer outcomes, breast cancer research

## Abstract

Introduction

Breast cancer is the most common cancer among women worldwide and one of the main causes of death in the female sex. Genetic polymorphisms in the mu-opioid receptor (OPRM1) and catechol-o-methyltransferase (COMT) genes have been shown to increase breast cancer risk. Variants in these genes may carry a prognostic impact in breast cancer. Long follow-up intervals are critical to adequately analyze prognosis in diseases with prolonged survival times and late relapses.

Objective

To analyze the impact of genetic polymorphisms on the survival of a cohort of breast cancer patients with very long follow-up.

Methods

This was a retrospective study of patients treated at Portuguese Oncology Institute of Porto (IPO Porto), a Portuguese comprehensive cancer center, with invasive carcinoma of the breast with very long follow-up, with analysis of genetic polymorphisms OPMR1 rs1799971 (AA vs. G allele) and COMT rs4680 (CC vs T allele) on biological samples. Statistical analysis of survival was performed using the Kaplan-Meier method, log-rank test, and Cox regression method.

Results

A total of 143 patients with invasive breast cancer were included, with a median follow-up of 21.5 years. There was a statistically significant difference in overall survival (OS) at 30 years according to the OPMR1 polymorphism, with lower survival in patients with the AA genotype (p<0.05). The difference in OS according to the COMT polymorphism was also statistically significant, with worse survival in patients with genotype T allele (p<0.05). The genetic variants were not associated with patient age, stage at diagnosis, or tumor grade.

Discussion

The genetic polymorphisms of OPRM1 and COMT affected the overall survival of breast cancer patients, in concordance with previous research. Further investigation is needed in order to clarify the prognostic impact of these genetic alterations on breast cancer.

## Introduction

Breast cancer is the most common type of cancer diagnosed in women worldwide, with 2.3 million new diagnoses in 2020. It still represents one of the main causes of death in females. In 2020, there were 7.8 million breast cancer patients globally, thus representing the most prevalent tumor in the world [1-2].

Prognostic definition in breast cancer is essential for the accurate choice of therapy and in order to inform patients’ expectations. Prognosis is the sum of clinical aspects such as patient age, lymph node status, and tumor size; histological features, namely, grade and lymphovascular invasion; division into molecular subtypes by hormone receptor status, HER2 status, and proliferative index (Ki67). More recently, tumoral genetic profiling has added prognostic information to traditional classifications [3].

The interaction between our genes and the environment is recognized as modulating many aspects of biology, but plenty of the implications of this interdependence are not yet identified. Several genetic alterations affect cancer susceptibility and may have predictive and prognostic value in cancer.

There are three subtypes of opioid receptors to which exogenous and endogenous opioids bind: mu, delta, and kappa. The mu-opioid receptor is fundamental in pain modulation, feelings of reward in drug abuse, and adverse effects of opioid drugs [4]. It is encoded by the gene OPRM1 found in chromosome 6q24-q25, of which the most common polymorphism is rs1799971 (locus A118G) [5]. The consequent substitution of asparagine to aspartate in position 40 of the receptor may change the signaling ability and expression in neural tissue. This single nucleotide polymorphism (SNP) has been linked to lower sensitivity to opioids in the population with the G allele, thus justifying higher analgesic dosing, especially in postoperative scenarios, increased opioid toxicity, and substance addiction [6-9]. Some studies also speculate that the G allele of this polymorphism is correlated with a higher breast cancer incidence [10].

The enzyme catechol-o-methyltransferase (COMT) plays a crucial role in the catecholaminergic system, metabolizing several fundamental substances, such as dopamine and estrogen, regulating pain perception [11]. Older studies have demonstrated a higher breast cancer incidence in women with lower COMT enzymatic levels [12]. The most prevalent polymorphism of the COMT gene is rs4680 (Val158Met), which is responsible for altered enzyme activity [13]. Studies on the polymorphisms of this gene have found a correlation with psychiatric disorders, Alzheimer’s disease, and fibromyalgia [14-16]. It has also been linked to the incidence of chemobrain in triple-negative breast cancer patients [17]. A meta-analysis confirmed an increased incidence of breast cancer in the Asian population with COMT polymorphism, despite inconsistent previous results [18-20]. Another study also found a correlation between COMT polymorphisms and breast cancer risk in postmenopausal women [21].

Little is known on how the individual genetic polymorphisms affect the oncologic outcome in breast cancer and the clinical relevance of key gene variants. The polymorphisms of the OPRM1 and COMT genes may influence survival and treatment response in this disease. A previous study found a lower mortality rate in patients with OPRM1 polymorphism in the G allele [22]. Prior research also reports a survival impact of COMT polymorphisms on patients diagnosed with breast cancer, thus conferring a prognostic significance to the genetic mutation [12,23].

Earlier detection and more effective treatments have increased the life expectancy in breast cancer patients. Long-term follow-up studies add valuable information on the survival of diseases with long overall survival and disease-free survival such as breast cancer. Additionally, late relapses of breast cancer are frequent, with relevant recurrence rates 10 years after diagnosis. Follow-up intervals of over 20 years may be key for establishing the prognostic factors in this disease [24].

The goal of the present study was to analyze the impact of these genetic polymorphisms on the survival of a cohort of breast cancer patients with a very long follow-up.

## Materials and methods

Population

A retrospective study was carried out, selecting consenting adult patients of a Portuguese comprehensive cancer center with histologically confirmed invasive carcinoma of the breast with over 12 years of follow-up (diagnosis between 1979 and 2009), with analysis of clinical records (paper and electronic). All patients were treated at the Portuguese Oncology Institute of Porto (IPO Porto), Portugal.

Exclusion criteria consisted of the absence of informed consent, lack of access to clinical records, and unavailability of biological samples.

Patients' clinical characteristics, data on treatments, response, relapse, and survival were obtained from medical records. The staging was made uniform through the American Joint Committee on Cancer (AJCC) 5th edition system, in accordance with the data available.

Biological samples and genotype selection

Peripheral venous blood samples of the patients were obtained using a standard technique and collected in ethylenediaminetetraacetic acid (EDTA)-containing tubes. Genomic DNA was extracted using the extraction kit Qiagen®, QIAmp DNA Blood MiniKit (Qiagen® 51106; Hilden, Germany), as indicated by the manufacturer’s procedure.

All samples were obtained with the informed consent of the participants prior to their inclusion in the study, according to Helsinki Declaration principles and after approval of the study by the Portuguese Institute of Oncology ethics committee (CES-IPO: 233/2017).

The most common genetic polymorphisms according to the available literature from OPMR1 and COMT were analyzed, namely, OPMR1 rs1799971 and COMT rs4680. Genotyping of the selected genetic variants was conducted using the TaqMan® Allelic Discrimination methodology in a real-time polymerase chain reaction (real-time PCR) system (Applied Biosystems, Waltham, Massachusetts). The procedures for real-time PCR reactions and amplifications were conducted according to the manufacturer’s protocol. To guarantee the quality of SNP genotyping, two negative controls were included in each amplification reaction preventing false positives and double sampling was conducted in at least 10% of the samples randomly chosen, with an accuracy above 99%. The genotyping results were individually validated by two researchers with no previous knowledge of the patient’s clinicopathological data.

Statistical analysis

Assessment of the association between genetic polymorphisms and patients’ clinicopathological characteristics was performed using the chi-square test (χ2) for categorical variables.

The overall survival (OS) and OS at 30 years were defined from the date of diagnosis to the date of death and the percentage of patients alive after 30 years of diagnosis, respectively. The disease-free survival (DFS) time was defined from the data from the date of diagnosis to the date of disease recurrence. Patients without disease relapse or those lost to follow-up were censored at their last date of record. Statistical analysis was carried out with SPSS software (IBM Corp., Armonk, NY). Analysis of survival was performed using the Kaplan-Meier method, log-rank test, as well as Cox regression method to calculate the hazard ratio (HR) and 95% confidence intervals (CI) for the association between the genotypes and risk of death, with adjustments according to previous treatment with endocrine therapy. A level of p<0.05 was considered statistically significant.

## Results

In this study, 143 patients were included, all adult women diagnosed with invasive carcinoma of the breast between 1979 and 2009, with a median follow-up of 21.5 years. Patient characteristics are described in Table 1.

**Table 1:** Patients' clinical and pathologic characteristics NR: Not Reported, NA: Not Applicable, AJCC: American Joint Committee on Cancer

Characteristics	n (%), total n=143
Age (years) Median: 48	
Follow-up (years) Median: 21.5 years	
Histology	
Invasive ductal carcinoma	123 (86.0)
Invasive lobular carcinoma	12 (8.4)
Other subtypes	8 (5.6)
Stage at diagnosis (AJCC 5^th^ Edition)	
I	26 (18.2)
II	62 (43.4)
III	31 (21.7)
IV	2 (1.4)
NR	22 (15.4)
Nodal involvement	65 (45.5)
Grade	
1	10 (7.0)
2	33 (23.1)
3	21 (14.7)
NR	79 (55.2)
HER-2 status	
Positive	7 (4.9)
Negative	27 (18.9)
NR	109 (76.2)
COMT rs4680 genotype	
CC genotype	35 (24.5)
T allele	95 (66.4)
NA	13 (9.1)
OPMR1 rs1799971 genotype	
AA genotype	6 (4.2)
G allele	127 (88.8)
NA	10 (7.0)
Neoadjuvant chemotherapy	4 (2.8)
Adjuvant chemotherapy	68 (47.6)
Adjuvant trastuzumab	3 (2.1)
Adjuvant radiotherapy	77 (53.8)
Adjuvant endocrine therapy	
None	50 (35.0)
Tamoxifen	47 (33.0)
Aromatase inhibitor	16 (11.2)
Tamoxifen – aromatase inhibitor	12 (8.4)
NR	18 (12.6)
Adjuvant endocrine therapy – duration (years) Median: 5 years	
Relapse	71 (50.0)
Local	18 (12.6)
Distance	48 (33.6)
Both	5 (3.5)

Tumor staging was compatible with T1 in 31%, T2 in 38%, T3 and T4 in 7% each, and not reported in 17%; 48% had no affected nodes and 46% were N+; nodal involvement was not reported in 6%. Tumor grade was G1 in 7%, G2 in 23% G3 in 14%, and not reported in 55%. Only 3% of patients received neoadjuvant chemotherapy; 48% of patients were submitted to adjuvant chemotherapy, of which 6% was based on anthracycline and taxane. Three patients with known HER-2-positive disease were treated with adjuvant trastuzumab. Adjuvant radiotherapy was carried out in 54% of patients. Endocrine therapy was not employed in 35% of patients, whereas 33% were treated with adjuvant tamoxifen, 11% with an aromatase inhibitor, and 8% with a combination of tamoxifen and a switch to an aromatase inhibitor.

Relapse was identified in 50% of patients in this cohort: 13% consisted of local relapse, 34% with distant metastasis, and 4% were diagnosed with both. Treatment was multimodal in most cases, including hormone therapy. To date, 57% of patients have died, 64% of which due to breast cancer. Median OS was 257 months (21.4 years), OS at 30 years was 51.7%. Median DFS was 239 months (19.9 years). There was no association between the genotypes and tumor grade, stage at diagnosis, or patient age at diagnosis (p>0.05).

**Figure 1:**
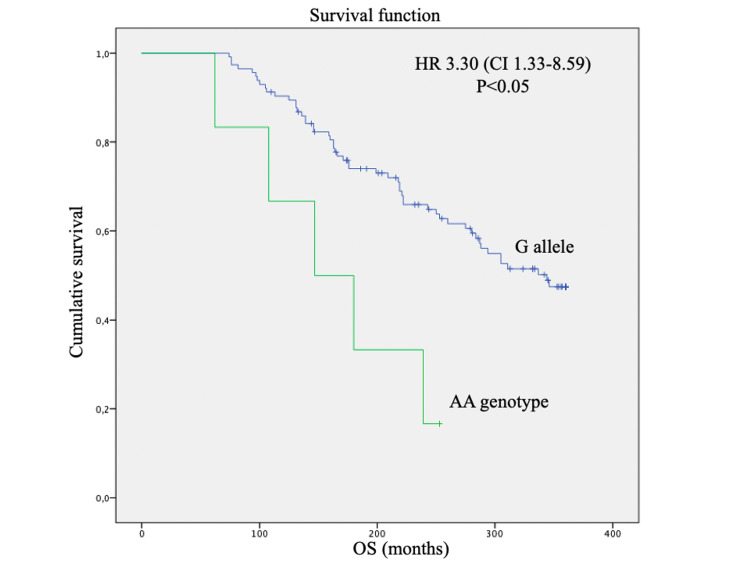
Overall survival by the Kaplan-Meier method, log-rank test, and Cox regression method of breast cancer patients according to OPRM1 rs1799971 polymorphism, log-rank test p=0.006 HR: Hazard Ratio, CI: Confidence Interval

In our study, there were no statistically significant differences in disease-free survival according to any of the polymorphisms (p>0.05). There was a statistically significant difference in OS at 30 years according to OPMR1 polymorphism, with lower survival in patients with AA genotype (p=0.006) and an HR of 3.30 (Figure 1). This difference was independent of adjuvant treatment with endocrine therapy.

**Figure 2:**
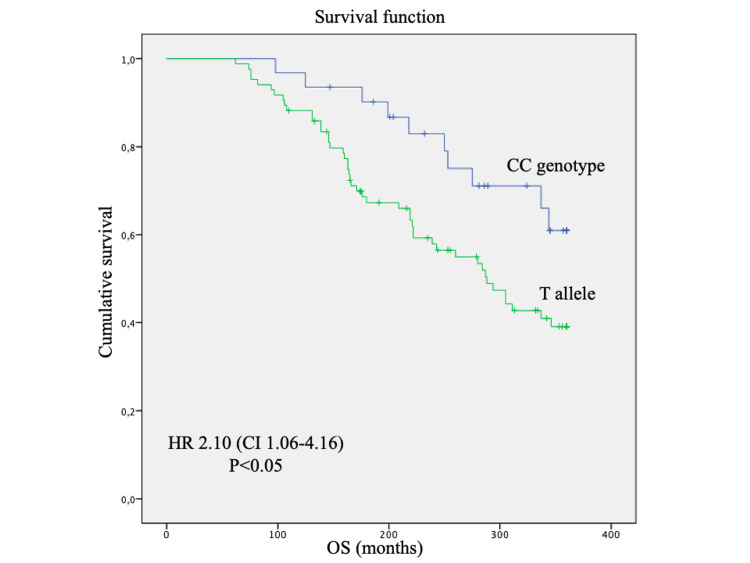
Overall survival by the Kaplan-Meier method, log-rank test, and Cox regression method of breast cancer patients according to COMT rs4680 polymorphism, log-rank test (p=0.03) HR: Hazard Ratio, CI: Confidence Interval

The difference of OS at 30 years according to the COMT polymorphisms was also statistically significant, with worse survival in patients with genotype T allele (p=0.03), with HR= 2.1 (Figure 2), independently of hormone therapy.

## Discussion

In our study, the genetic polymorphisms of OPRM1 and COMT affected the overall survival of breast cancer patients. The AA genotype for OPRM1 conferred a 3.3 times higher risk of death at 30 years and the presence of the T allele increased the risk of death by 2.1 times at 30 years, regardless of adjuvant hormone therapy. The genotypes did not influence patient age, stage at diagnosis, or tumor grade. These findings demonstrate the current extent of our ignorance on the prognostication of breast cancer, despite all the previous advances.

Due to the increasing survival rates of breast cancer patients, longer follow-up studies are required to shed light on prognostic data. Our paper represents a unique study that travels back in time through biological samples and unveils data on patients with long follow-up times. Real-world data of different geographic locations are necessary in order to fully grasp the implications of genetic variants in diverse populations. Our study population, of European ancestry, contrasts with a predominance of genetic studies of these polymorphisms on patients of Asian descent.

The present result is consistent with a previous paper describing the survival impact of the OPRM1 rs1799971 genotype in a sample of over 2000 women [22]. Opioids and opioid receptors may be directly involved in intratumoral signaling for proliferation, apoptosis, and the ability for invasion; opioid receptors may also have indirect effects on cancer due to immunosuppression and angiogenic modulation properties [24-25]. The immunosuppressant effects of opioids, mediated by the mu-opioid receptor, have been described in intravenous drug users [26].

Prior research confirms the effect on the survival of the COMT rs4680 polymorphism, in accordance with the present study [13,21]. Matsui et al. describe a correlation between COMT genotype and lymph node metalization and clinical stage, which was not apparent in our sample [27]. Lower estrogen degradation may lead to higher exposure to endogenous compounds and eventually lower therapeutic efficacy of endocrine therapy. On the other hand, it has been postulated that estrogen metabolites may cause cellular injury through indirect mechanisms, including oxidative stress and DNA damage [12,28-29].

There is a growing interest in the characterization of genetic alterations in the oncologic population, paving the way for personalized cancer treatment. Prognostic scores could be developed to guide the tailoring of therapy to individual risk and according to enzymatic levels of key metabolizers such as COMT.

Currently, staging, endocrine receptor and HER-2 status, and Ki-67 and tumor grade define adjuvant treatment choices, occasionally supported by genetic signature tools such as OncotypeDx and Prossigna. This study reminds us of the potential of analyzing patient characteristics instead of tumoral features. Successful cancer treatment is certainly dependent on the interactions between the tumor, host, and environment; however, little advances have been made in this field.

Our main limitations are intimately associated with the problems of retrospective studies. Moreover, we ought to mention the heterogeneity of our study population, which derives from the historic nature of this retrospective study - the evolution of staging and tumoral biomarker identification and treatment is evident. In order to diminish this potential bias, we restaged all patients with the same system, the AJCC 5th Edition of 1997, which was compliant with the lack of modern biomarker data at the time of diagnosis. The availability of biological samples of women treated decades ago was also a constraint for patient selection and population size. Bio-banks were not common in the past, therefore, we count ourselves fortunate to have these archived samples accessible for investigation.

Multicentric, international, retrospective, and prospective studies on this area of research are needed to expand the new perspectives on cancer survival this study has unlocked.

## Conclusions

In the present study, OPRM1 and COMT polymorphisms demonstrated a prognostic impact in breast cancer, significantly affecting overall survival at 30 years. Genetic variants were not associated with age, staging, or tumor grade, thus potentially constituting a new independent prognostic variable. This knowledge could aid to individualize oncologic treatment according to each patient's genetic risk.

Further research is needed, preferably with prospective trials, in order to clarify the present results. The importance of broad inter-institutional bio-banks must be underlined, to allow future investigation in this budding area.
